# The effect of breast cancer awareness month on internet search activity - a comparison with awareness campaigns for lung and prostate cancer

**DOI:** 10.1186/1471-2407-11-442

**Published:** 2011-10-12

**Authors:** Ronan W Glynn, John C Kelly, Norma Coffey, Karl J Sweeney, Michael J Kerin

**Affiliations:** 1 Department of Surgery, National University of Ireland Galway, Ireland; 2 Biostatistics Unit, Clinical Research Facility, National University of Ireland Galway, Ireland

## Abstract

**Background:**

This work aimed to assess the effects of the annual breast cancer awareness campaign on internet search activity, and to compare these effects with those of similar campaigns in prostate and lung cancer. We further aimed to assess overall levels of online activity relating to all three neoplasms between 2004 and 2009.

**Methods:**

*Google Insights for Search *was employed to examine search trends for the term "breast cancer", across all Google domains between January 2004 and December 2009 (6 years). Search trends for both "prostate cancer" and "lung cancer" across all domains were also analysed for the same period, and these trends were compared with those for "breast cancer". Repeated measures ANOVA and Tukey post-hoc analyses were performed to assess for significant differences in activity.

**Results:**

Increased levels of online activity relating to breast cancer are consistently generated each October. There is a significantly higher level of background activity in breast cancer compared with that in lung or prostate cancer (p < 0.001), and the October campaign stimulates online activity more effectively than equivalent campaigns for these other malignancies (p < 0.001).

**Conclusions:**

The annual breast cancer awareness campaign is proving effective in stimulating online activity and may hold useful lessons for other cancer awareness initiatives.

## Background

Breast Cancer Awareness Month (BCAM) is an international health campaign organised each October in order to raise awareness of the disease, and to raise funds for ongoing research. The campaign, which celebrated its 25th anniversary in the United States in October 2009, is characterised by an effort to underscore the importance of self-examination and screening, whilst promoting existing resources which can assist those motivated by the campaign to adopt these behaviours[1]. While there are dissenting voices regarding the amount of attention which breast cancer receives as a result of these, and other breast awareness initiatives[2,3], it is accepted that these campaigns have improved care for patients by enabling better prevention, screening, knowledge and understanding of treatment options, research funding, and political will[3].

In tandem with the development of BCAM has been the growth and evolution of the Internet. Today, over 80% (113 million individuals) of all American Internet users employ the Internet to access health information[4] and those who use the Internet to search for information regarding a personal health problem are 60% more likely to contact a health professional compared with those who have not searched online[5]. In relation to cancer specifically, it has been demonstrated that use of the internet as a source for oncological information is increasing rapidly, with one recent study demonstrating utilisation by 63% of cancer patients[6]. These developments have not gone unnoticed by advocates seeking to raise awareness of particular cancers, as demonstrated by the ubiquity of advocate-affiliated websites now available online. Given the apparent relationship between people's online activity and health behaviour, and the increasingly omnipresent influence of the Internet in daily life, this study aims to examine the effects of BCAM on the Internet habits of the American population. We aimed to do this by examining search trends for the phrase "breast cancer" in Google, using the recently developed *Google Insights for Search Application *on the internet[7]. This application has previously been demonstrated to examine public interest in *in vitro *fertilisation[8], and to help predict the development of influenza epidemics[9], and outbreaks of salmonella[10], chickenpox and gastroenteritis[11], by tracking health-seeking behaviour. In addition, we aimed to examine search interest in both prostate and lung cancer, and to compare online activity for these malignancies with that of breast cancer.

## Methods

The *Google Insights for Search Application *is a search-volume reporting tool which provides aggregated data from January 2004. The tool only shows results for search terms that receive a significant amount of traffic, and enforces minimum thresholds for inclusion. Data is normalised to the reference population, in this case the United States, and scaled from 0-100. The system designates peak search activity over a given time period as 100, and activity at all other times is then presented relative to that peak. The relative search volume may be interpreted as the probability that a random user searched for a particular search term from a specific location and time. It should be noted that a downward trending line doesn't necessarily mean that the absolute traffic for a search term is decreasing - only that its popularity (or query share) is decreasing. Query share can be understood as the ratio between the number of queries for that term and the total number of queries (at a given time and location)[7].

We examined search trends for the term "breast cancer", across all Google categories between January 2004 and December 2009 (6 years) inclusive. Search trends for both "prostate cancer" and "lung cancer" across all categories were also analysed for the same period, and these trends were compared with those for "breast cancer". Following appraisal of related terms for each cancer, the search terms "breast cancer", "lung cancer" and "prostate cancer" were chosen because these returned the greatest volume of search activity for each of the cancers under study,

Data points consisted of repeated measurements of search activity, one for each week in the year over 6 years. To assess if there were any significant changes in average search activity from month to month and year to year, repeated measures ANOVA was used. Each week was assigned to a month, e.g. weeks 1-4 were assigned the month January, and so on. In weeks where there was an overlap in months, (i.e. one month ended and the second month began in the same week), the week was assigned to a month if more than 4 days in the week belonged to that month (i.e. if a week contained the last 3 days in August and first 4 days in September it was assigned to September). Since the number of observations for each month varied between 4 and 5, this design was unbalanced and a general linear model was used to fit the ANOVA model. Two factors were examined; the first tested for differences in average search activity by year (year factor) and the second tested for differences in average search activity by month (month factor). The ANOVA table was calculated using year and month as fixed factors and the results indicated whether or not there were significant differences in average search activity between months and/or years. Where significant differences were identified, Tukey post-hoc tests were performed to determine between which months/years these differences existed. All of the analysis was carried out in MINITAB.

In order to identify which search terms generated the highest levels of activity in their respective awareness months, a further search was performed for the three months concerned in 2009; the top searches for the time periods are returned as part of the *Google Insights for Search Application *analysis.

## Results

**Figure 1:**
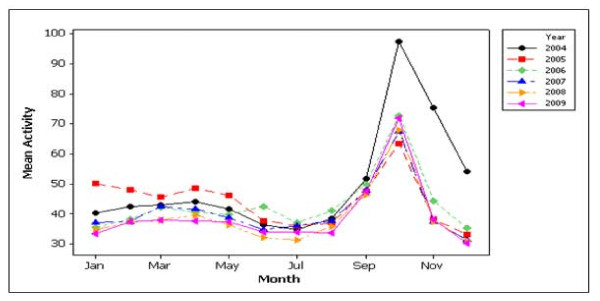
**Scatter plot for mean activity on breast cancer versus month**. Peak search activity was recorded in October 2004, and all other data is normalised relative to this peak.

**Figure 2:**
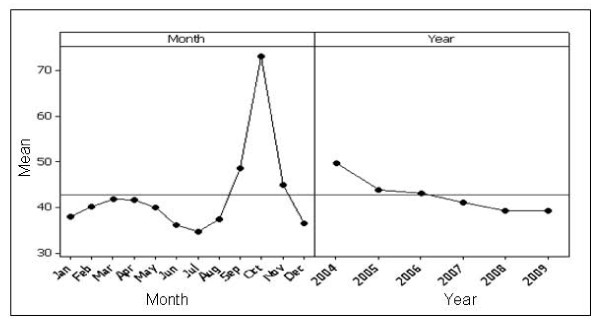
**Main effects plot for breast cancer**. The plot on the left demonstrates search activity for each month, where each point is the average of all values for a particular month across all years, e.g. point 1 is the average search activity for January across all years. The plot on the right shows the effect of year, where each point is the average search activity for a particular year.

Figure 1 shows the plots of mean search activity for breast cancer by month, whilst Figure 1(b) shows a main effects plot for breast cancer. Mean search activity was significantly higher in October versus all other months (p < 0.001). There was a significant increase in search activity from August to September and from September to October, followed by significant decreases in search activity from October to November and November to December (all p < 0.001) (Figure 2). There was a significant decrease in search activity from 2004 to 2005, but no significant changes in search activity from 2005 to 2006, 2006 to 2007, 2007 to 2008 and 2008 to 2009. The higher levels in 2004 were generated as a result of particularly high levels of activity in October, November and December of that year.

**Figure 3:**
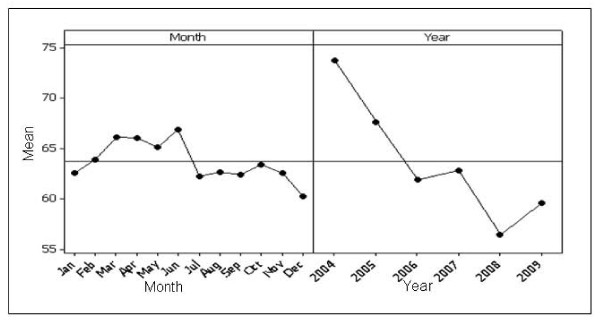
**Main effects plot for prostate cancer**. The plot on the left demonstrates search activity for each month, where each point is the average of all values for a particular month across all years, e.g. point 1 is the average search activity for January across all years. The plot on the right shows the effect of year, where each point is the average search activity for a particular year.

Figure 3 shows a main effects plot for prostate cancer. There were no significant differences in mean search activity when comparing prostate cancer awareness month (September) to all other months. There were also no significant differences when comparing consecutive months. Mean search activity decreased overall from 2004 to 2009, although there was a significant increase in activity from 2008 to 2009 (p < 0.001).

**Figure 4:**
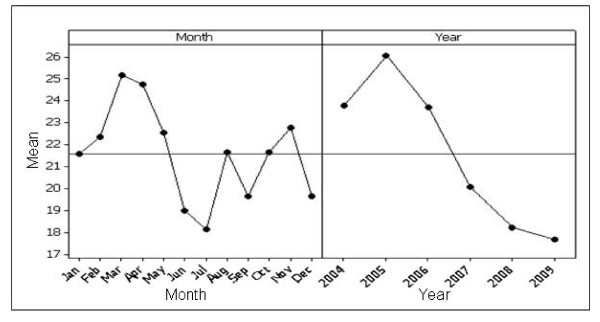
**Main effects plot for lung cancer**. The plot on the left demonstrates search activity for each month; each point is the average of all values across all years, e.g. point 1 is the average search activity for January across all years. The plot on the right shows the effect of year; each point is the average search activity for a particular year.

Figure 4 shows a main effects plot for lung cancer. There were no significant differences in mean search activity between lung cancer awareness month (November) and other months. Mean search activity demonstrated a decreasing trend through 2004 to 2009.

**Figure 5:**
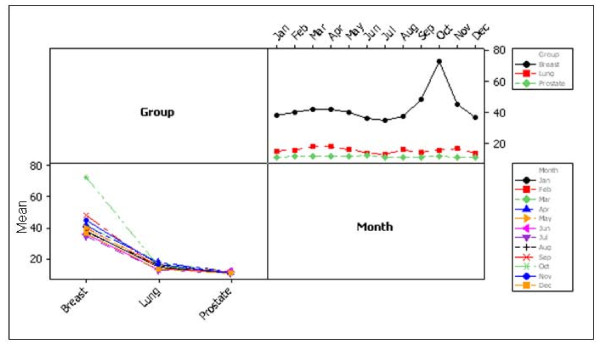
**Interaction plot for cancers by year**. The plot on the left demonstrates search activity for each of the three cancers; each point is the average search activity for a particular year. The plot on the right shows search activity by month; each point represents an average of all values for a particular month across all years.

Figure 5 demonstrates comparative search activity for breast, prostate and lung cancer. Breast cancer search activity was significantly higher than lung cancer activity (p < 0.001) and lung cancer activity was significantly higher than prostate cancer activity (p < 0.001) (Figure 5, Top right). Comparing activity levels across years revealed significantly higher average search activity levels in breast cancer versus prostate and lung cancer for all years (p < 0.001) (Figure 5, Top right). There were also significantly higher levels of lung cancer activity in 2004, 2005 and 2006 than prostate cancer (p < 0.001) (Figure 5, Top right). On average breast cancer research activity was significantly higher in every month of the year versus the other two cancers (p < 0.001) (Figure 5, Bottom left).

Table 1 demonstrates the top searches for each of the cancers under study during their respective awareness months in 2009. Just two and five search strategies generated sufficient activity to permit analysis by the *Google Insights for Search Application*, for prostate and lung cancer, respectively.

**Table 1:** Top 10 search terms for each of the cancers under study during their 2009 awareness month

Rank	Breast Cancer (October 2009)	Prostate Cancer (September 2009)	Lung Cancer (November 2009)
1	Breast cancer awareness	Prostate cancer symptoms	Lung cancer symptoms
2	Breast cancer walk	Prostate cancer treatment	Obama lung cancer
3	Breast cancer month		Lung cancer treatment
4	Pink		Lung cancer prognosis
5	Breast cancer ribbon		Lung cancer stages
6	Cancer symptoms		
7	Breast cancer symptoms		
8	Susan komen		
9	Breast cancer nfl		

## Discussion

It has been demonstrated that limited cancer awareness amongst the general public can result in delayed presentation and poorer survival [12,13]. BCAM has been attempting to address this problem in breast cancer since 1985, and has been demonstrated to result in an increased uptake of screening mammography[14], and an increased rate of detection of *in situ*- and local breast tumours[1]. The results presented here suggest that the BCAM campaigns have been highly successful in stimulating Internet activity relating to breast cancer, at least for the duration of the annual campaign, and for the months immediately either side of it.

We have found that breast cancer is responsible for a much higher level of search activity overall when compared with both prostate and lung cancer. This result correlates well with previous work which examined cancer search activity using the Yahoo! search engine between 2001 and 2003; breast cancer ranked first of 23 cancers in terms of search activity, ahead of lung cancer in second and prostate cancer in fifth[15]. The most likely explanation for these findings is that breast cancer (number affected United States 2006, 2,605,000) has a greater prevalence than either prostate (n = 2,320,000) or lung cancer (n = 426,000)[16]. One further explanation is that American women have been shown to overestimate their breast cancer risk[17], and thus may have a lower threshold for searching out information and advice regarding breast cancer. However, our analysis demonstrating that the September and November campaigns to promote awareness of both prostate and lung cancer, respectively, have not increased Internet user activity relative to the rest of the year is more difficult to explain. In addition, it is clear from Table 1 that the campaigns for these latter cancers do not appear to register in the public mindset, with neither campaign nor their associated awareness initiatives achieving sufficient interest to register on the *Google Insights for Search Application*. In contrast, it is clear that the strategies employed by BCAM result in online activity, with high levels of interest demonstrated in relation to both the campaign and its associated initiatives ('breast cancer walk', 'breast cancer NFL'), and indeed to the disease itself ('breast cancer symptoms').

As noted in the introduction to this work, there has been an increasing level of disquiet at both the success of BCAM, and perhaps more specifically with the 'pink ribbon' culture which has developed around breast cancer; there are now 1468 major non-profit organisations involved in its promotion in the United States, compared with just 229 and 154 organisations for prostate and lung cancer, respectively[18]. An argument, too, has been made that BCAM, and pink culture in general, now serves simply as the official platform for an industry led promotional campaign aimed at maintaining a competitive edge in the marketplace[19]. From a clinical standpoint, although the campaign has been attributed with positively influencing the diagnosis and management of breast cancer, recent work by Jacobsen et el. has questioned its ongoing role in raising awareness; the authors examined 30 years of registry data to determine if October events relating to BCAM lead to increases in the following month of November - no increases were seen[20]. The authors concluded that, in contrast to the earlier years of the campaign, "more recently, the increase in routine screening has contributed to a decrease in the impact of specific promotion events on new diagnoses"[20]. Of course, whilst much of the money raised via BCAM is directed towards aspects of breast cancer out-with diagnosis and screening, the apparent undue attention which BCAM, and indeed breast cancer in general, continues to receive has raised concerns amongst other advocacy groups. Their misgivings are based on the belief that the attention being given to breast cancer, in terms of both research time and money, is leading to a concomitant neglect of research into other malignancies. One such group is the National Prostate Cancer Coalition (NPCC) in the United States. They have pointed out, for example, that in 2006, research spending by the National Cancer Institute (NCI) on breast cancer exceeded 718 million dollars whilst prostate cancer was allocated just 376 million[21].

Given this expenditure, and indeed the significantly greater research output associated with breast cancer [22], it is all the more important that the success or otherwise of awareness initiatives for both prostate and lung cancer are scrutinised such that weaknesses may be addressed. In an era of ever increasing Internet use, the failure of awareness campaigns to register significant levels of online interest must raise concerns for those involved. An argument could be made that the differences discussed above relate to the patient demographics associated with each of these malignancies. In particular, it has previously been demonstrated that women are more likely to seek health-related information on the internet than are their male counterparts [23,24]. In addition, women overestimate their risk for breast cancer[25], and increased perceived cancer risk has been demonstrated to correlate with increased health-seeking behaviour online [26]. Furthermore, it might be argued that, since levels of Internet activity have been shown to be positively correlated with higher socioeconomic status and education level, it might well have been expected that lung cancer in particular, which has a higher disease burden in lower socioeconomic groups [27], would consequently be associated with decreased Internet activity. Similarly, lung cancer tends to affect an older cohort of patients, relative to the other two malignancies, and is not associated with a readily available screening test, and again these differences might well account for some of the differences seen.

These arguments notwithstanding, recent studies have reported that 56-58% of patients with prostate cancer [28,29], and 68% of those with known or suspected lung cancer access health information on the Internet[30]; this compares with some 48-50% of those with breast cancer [31,32]. In addition, the most recent report on this subject found no differences in Internet use according to age, education level and economic status in a cohort of patients with breast or colorectal cancer, and concluded that the main reason for this was "due to the increased availability of the Internet and a decrease in the cost of computers and Internet access, making economic factors less influential"[33].

The aforementioned ubiquity of Internet use across the different cancer types suggests that the success of BCAM in increasing Internet activity, relative to the success of similar campaigns for either prostate or lung cancer, may be attributed to characteristics of the campaign itself, rather than to characteristics of the target population. Cooper *et al*. have reported that levels of cancer-related search activity on Yahoo! demonstrated significant positive correlation with levels of news coverage (p < 0.001)[15]; breast cancer has been repeatedly shown to receive more attention than any other malignancy in both the print[34] and television media[35], and we suggest that the increased online activity seen during BCAM is directly consequent on the success of the campaign in focusing media attention upon it, as exemplified by it's promotion on the front cover of an October 2009 edition of *Sports Illustrated*. This successful marketing campaign has, as noted, aroused some degree of unease amongst advocates for other causes however. In addition, whilst the development of BCAM and "pink culture" has had undeniable benefits for the breast cancer movement in general, an argument can also be made that the increasing ubiquity of breast cancer in daily life has had deleterious consequences for some patients, with the focus on lifestyle modification and risk factor avoidance leading some to feel guilty in relation to their past behaviour[2]. In addition, some have questioned whether the success of campaigns have led to a situation whereby women are now unable to make an informed decision as to whether or not they should attend for screening, with very little public commentary being given to the potential downside of this attendance[36]. Concerns have also been raised regarding the statistics employed in campaigns; whilst the UK literature, for example, claims that one in nine women will suffer breast cancer at some time in their lives, the reality is that many of these will be diagnosed in the elderly who will die of some other cause[2]. In shaping future initiatives, then, it will be important for agencies working on behalf of other cancer groups to address these issues within their own sphere such that similar concerns are not raised in relation to their efforts to promote cancer awareness.

One important caveat to this work is that whilst it has demonstrated the apparent success of BCAM compared to other cancer awareness initiatives in raising levels of related online activity, this finding cannot be extrapolated to conclude that this increased activity correlates necessarily with either increased cancer awareness or health seeking activity offline. As alluded to in the introduction, there is some evidence to suggest that online activity is associated with health seeking behaviour[5]. This relationship is complex however, and whilst Ybarra et at reported that 55% of online health information seekers contacted a health professional as a result of the information they found online, the authors qualified this by noting that the subgroup who used the Internet to access health information because it was free, or because seeing a health professional was expensive, were 90% less likely to contact a healthcare professional as a result of their online activity[5]. In addition, the 2005 Health Information National Trends Survey reported that while over half of respondents aged between 18 and 34 years old, and almost 40% of those aged between 45 and 64 years, preferred going online first to obtain information about cancer, doctors nevertheless remained the most trusted source of health information[37]; this finding correlates well with the results of a 2011 study of younger men with prostate cancer, wherein doctor's advice about treatment strategies superceded the Internet in influencing behaviour [38]. Whilst this latter work is just one of a growing number of studies which have examined the use of the Internet amongst cancer patients and cancer survivors[39], there remains a dearth of work examining the influence of online activity in raising awareness of cancer risk and stimulating offline health behaviour prior to receiving an actual cancer diagnosis; it therefore remains to be demonstrated conclusively that the increased levels of online activity associated with BCAM shown in this study lead to increased uptake of either screening or cancer avoidance strategies. Thus, although one might intuitively predict that increased online activity will impact positively on timely cancer diagnosis and prevention, it is too early to conclude that the success of BCAM in stimulating online activity may be taken as a proxy for concluding that BCAM raises awareness of, or motivates offline activity in relation to avoidance of breast cancer.

There are a number of other limitations to this work. As noted in similar work examining search interest in *in vitro *fertilisation, analysis of internet activity is necessarily limited to those with online access, and using Google search engines[8]. In addition, it is not possible to identify which stakeholders (i.e. advocates, patients, health professionals, etc.) are responsible for the search activity. This has particular significance given the aforementioned commercialisation of breast cancer and the development of "pink culture"; it may be that many of those seeking information online as a result of BCAM are in fact already allied with this movement, either as a result of a personal or family history of breast cancer, or through their work or involvement in non-profit or private organisations linked to breast cancer. If this were the case, it would raise questions as to whether the increased levels of online activity demonstrated here actually represent success in terms of targeting the population at risk, thereby achieving success as defined by the BCAM organisation itself - the education and empowerment of "women to take charge of their own breast health by practicing regular self-breast exams to identify any changes, scheduling regular visits and annual mammograms with their healthcare provider, adhering to prescribed treatment, and knowing the facts about recurrence"[40]. Finally, the *Insights for Search Application *normalises and scales data between 0 and 100 - actual search figures are not provided, and therefore the magnitude of the differences in Internet search activity discussed above cannot be elaborated upon.

## Conclusion

The above limitations notwithstanding, this report provides an overview of overall online activity concerning three common malignancies. It seems reasonable to conclude that aspects of the BCAM promotional effort, and in particular the degree to which this effort has managed to attract media attention, has been successful in increasing Internet activity relating to breast cancer. Given the degree to which this medium is now employed in accessing information, shaping opinion and motivating offline activity[5], there are perhaps lessons - both in terms of what works, but also in terms of what is best avoided - to be learned from the BCAM campaign which might usefully be adapted for other cancer awareness initiatives, whilst simultaneously providing useful information for breast cancer advocates and supporters alike.

## List of Abbreviations

BCAM: Breast Cancer Awareness Month; ANOVA: Analysis of Variance

## Competing interests

The authors declare that they have no competing interests.

## Authors' contributions

All authors made substantial contributions to the conception and design, or acquisition of data, or analysis and interpretation of data; all have been involved in drafting the manuscript or revising it critically for important intellectual content; and all have given final approval of the version to be published. Each author should have participated sufficiently in the work to take public responsibility for appropriate portions of the content.

## Pre-publication history

The pre-publication history for this paper can be accessed here:

http://www.biomedcentral.com/1471-2407/11/442/prepub
